# Trends and Disparities in Quality of Life Among Older Adults From 1998 to 2018 in China: A National Observational Study

**DOI:** 10.3389/fmed.2021.796208

**Published:** 2022-01-28

**Authors:** Jue Liu, Jun Wang

**Affiliations:** ^1^Department of Epidemiology and Biostatistics, School of Public Health, Peking University, Beijing, China; ^2^Institute for Global Health and Development, Peking University, Beijing, China; ^3^Center for Health Policy Research and Evaluation, Renmin University of China, Beijing, China

**Keywords:** quality of life, prevalence, observational study, older adults, China

## Abstract

**Objective:**

To investigate 20-year trends and disparities in quality of life among older adults in China from 1998 to 2018.

**Methods:**

Our study was based on eight representative nationwide health surveys among older adults conducted in China from 1998 to 2018. Quality of life data were obtained from 91,993 individuals aged 65 years or above. All surveys included identical indicators of self-reported quality of life, demographic factors, socioeconomic status, lifestyle habits, and health status. The trends in the standardized prevalence of poor quality of life from 1998 to 2018 were examined by locally weighted scatterplot smoothing regression (LOWESS) analysis. We assessed the prevalence of poor quality of life and its related factors by logistic regression models after adjusting for potential confounders.

**Results:**

The prevalence of poor quality of life was 38.2% (95% *CI*: 37.9–38.5%). The trends of poor quality of life showed an inverted “U” shape, that the prevalence increased from 27.8% in 1998 to 43.6% in 2008, and then decreased from 39.2% in 2011 to 32.1% in 2018. Disparities in the prevalence of poor quality of life were exacerbating among participants with low or moderate household income per capita and participants with high household income per capita from 1998 to 2018. After controlling potential confounders, living in rural areas, aged below 80 years, unmarried, living alone, low household income, current smoker, poor dietary diversity, never participating in organized social activities, with chronic diseases, functional disability, poor self-reported health, and unhealthy psychological status were risk factors related with poor quality of life in the multivariate model (all *p* < 0.05).

**Conclusion:**

During the past two decades, poor quality of life in Chinese older adults showed an inverted “U” trend from 1998 to 2018 that the prevalence of poor quality of life peaked in 2008 and declined since China's deepening health system reform in 2009. However, disparities in the poor quality of life were exacerbating among participants with different socioeconomic statuses. Strengthening the health system is of great importance in improving the quality of life. More efforts are needed to reduce the disparities in the quality of life among the population at the different socioeconomic levels.

## Introduction

Ensuring healthy lives and promoting well-being for all at all ages by 2030 is a global sustainable development goal (SDG) set by the United Nations. Monitoring the health status of populations is crucial for recognizing unmet population health needs, planning intervention programs, and assessing the effectiveness of health policies and strategies (1). Among all the indicators on health, quality of life is considered as a multi-dimensional and comprehensive indicator that better reflect well-being, which is affected by physical, psychological, and social factors (2, 3). Numerous studies had explored age, residence, income level, lifestyle factors, chronic disease, and social health status as factors associated with quality of life (2, 4–8).

Some studies assessed the time trends of poor quality of life (1, 6, 9, 10). However, the results were controversial because of different populations, assessment tools, locations, and periods. Audureau et al. (1) reported evidence of worsening trends and increasing demographic, socioeconomic, and regional disparities in quality of life by comparing two French population-based cross-sectional surveys in 1995 and 2003. Atlantis et al. (6) investigated the 10-year trends in quality of life among 9,059 people aged ≥15 years who participated in representative surveys of the South Australian population in 1998, 2004, and 2008. They found that scores on the physical component of SF-36 were stable and scores on the mental component were significantly decreased from 1998 to 2008 (6). Rehkopf et al. (10) found that general health improved in people aged 65 years and older in the United States from 2003 to 2017 with the percentage of poor health decreasing from 23% in 2003 to 19% in 2017.

Literature had reported the potential factors associated with poor quality of life in the general population or patients in China (3, 8, 11, 12). Zhang et al. (12) found that the prevalence and decline in quality of life of multimorbid older-aged people were severe in Shandong province, China. Aging is one of the global challenges leading high economic burden on health and social care (13). China has the largest number of older adults in the world, imposing a heavy burden on the healthcare systems. There are more than 260 million older adults in China and the proportion of people aged 60 and older continues to increase in the past decade, according to the 17 National Census in 2020. China has deepened health system reform since 2009, to reach the goal of achieving universal health coverage by 2020. Understanding the trends and disparities in quality of life among older adults in China is helpful for recognizing the achievements and gaps of health services and needs, and better making tailored interventions, strategies, and policies. However, studies examining the long-term trends of quality of life were limited in China (14). To the best of our knowledge, this is the first study that focuses on 20-year trends and disparities in quality of life among older adults in China that reflect the effect of China's deepening health system reform in 2009, which is different from previous studies made with the Chinese Longitudinal Healthy Longevity Survey (CLHLS). The study is unique due to the potential multiculturalism of the findings and its large sample size.

In the present study, we aimed to investigate the trends and disparities in quality of life among older adults aged 65 years and older in China from 1998 to 2018, using the national cross-sectional data from eight representative health surveys among older adults conducted in China in 1998, 2000, 2002, 2005, 2008, 2011, 2014, and 2018.

## Methods

### Study Population and Data Source

This is a national observational study using data from the CLHLS. The CLHLS aimed at investigating the determinants of healthy longevity among the older Chinese population and covered 22 of 31 provinces in China (15, 16). The survey was conducted randomly in about half of the cities/counties in 22 out of 31 provinces in China, covering about 85% of the national population. It began in 1998 and continued in 2000, 2002, 2005, 2008, 2011, 2014, and 2018, with about a 90% response rate for each wave (17). Nearly one-third of participants from each wave were from the previous wave, and the rest were new recruits because of the mixed longitudinal design of CLHLS. To reduce the selection bias in different waves and ensure the consistency of the study population, new recruits were selected based on the similarities in gender, age, and general characteristics with those who were lost during the follow-up. More details of the CLHLS study design can be found elsewhere (15–17).

**Figure 1:**
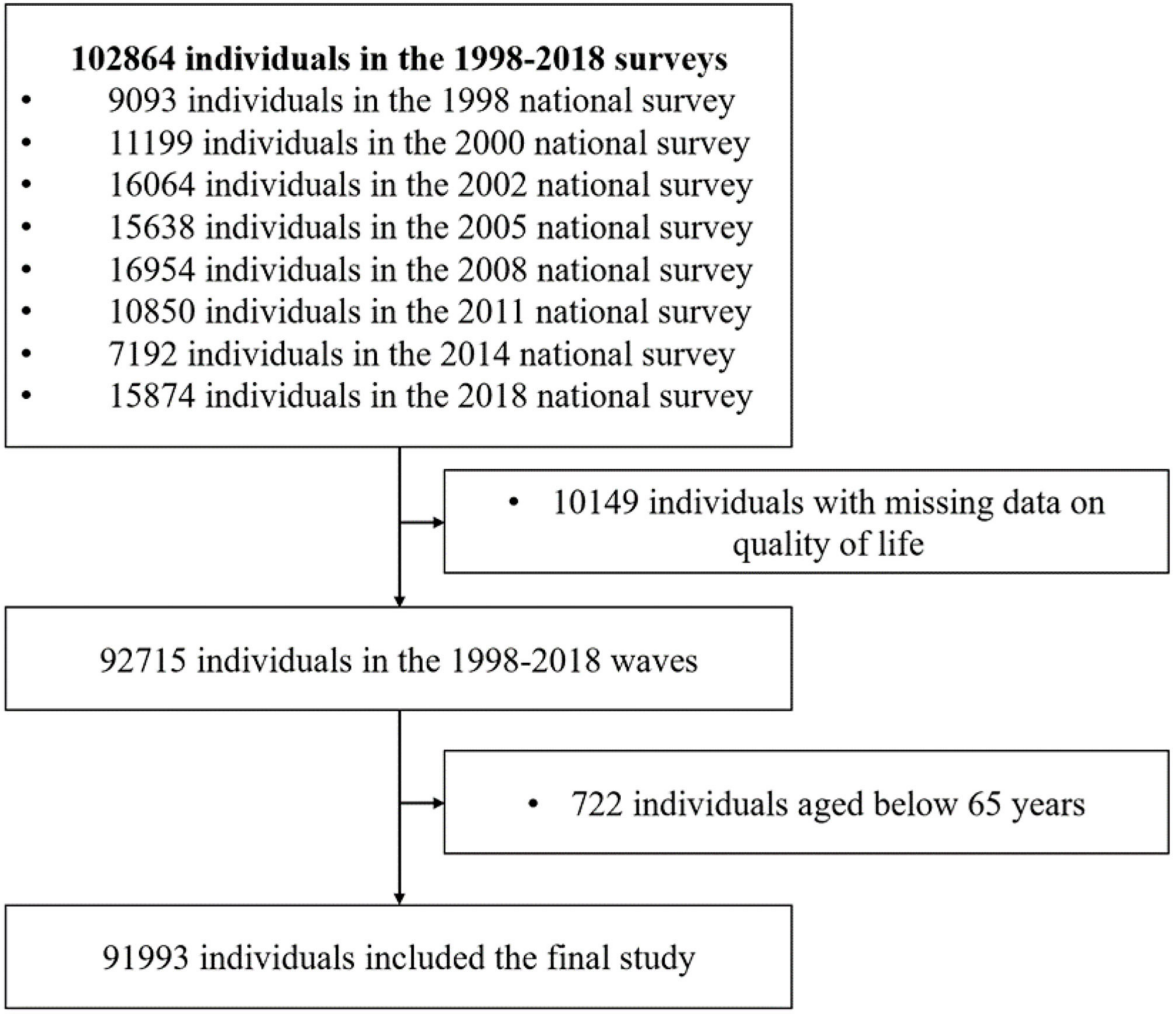
Study profile.

There was a total of 1,02,864 participants in these nine waves (9,093 in 1998, 11,199 in 2000, 16,064 in 2002, 15,638 in 2005, 16,954 in 2008, 10,850 in 2011, 7,192 in 2014, and 15,874 in 2018). Among them, we excluded 10,149 participants who had missing data on quality of life and 722 participants aged below 65 years, yielding 91,993 participants (89.4%) in the final study (Figure 1).

### Quality of Life

All information was obtained in the homes of participants through face-to-face questionnaire interviews and physical health examinations by trained investigators. Quality of life was assessed by asking one question as “How do you rate your quality of life at present? (Very good, good, so so, bad, very bad)”. We combined the answer “so so,” “bad,” and “very bad” as self-reported poor quality of life.

### Explanatory Variables

Following previous studies (17–20), we included explanatory variable groups derived from the CLHLS in this study, such as wave indicators (investigation years), demographic factors, socioeconomic status, lifestyle habits, and health conditions variables. Demographic factors included region (urban or rural), gender (male or female), age group (65–79 years or ≥80 years), marital status (unmarried, married, or divorced or widowed), and living patterns (living with family members, living in an institution, or living alone). Socioeconomic status included years of schooling (0 years or ≥1 years), household income per capita (low, moderate, or high), and frequently going to bed hungry in childhood (yes or no). To make the household income of different years comparable, we divided the household income of each wave into three groups by tertiles according to the level of each survey year. Lifestyle habits included smoking status (never, previous, or current), alcohol intaking status (never, previous, or current), regular exercise (never, previous, or current), dietary diversity (poor, moderate, or good), participating in organized social activities (almost every day, sometimes, or never). Dietary diversity was evaluated as poor (0–3), moderate (4–6), or good (7–9) by the calculated dietary diversity score (0–9) reflecting the consumption numbers of nine types of food groups (meat, vegetables, fish, eggs, fruits, legumes, milk, tea, and nuts) (21). Health status included body mass index (BMI) (underweight, normal weight, overweight, or obesity), numbers of chronic diseases (0, 1, or ≥2), functional disability (no or yes), cognitive impairment (no or yes), self-reported health (good, general, or poor), psychological status (healthy or unhealthy). BMI was categorized as underweight (<18.5 kg/m^2^), normal (18.5–24.9 kg/m^2^), overweight (25–29.9 kg/m^2^), and obese (≥30 kg/m^2^), according to the WHO. Functional disability was defined as the self-reported difficulty with any of the following activities of daily living (ADL) items, such as dressing, eating, bathing, continence, toileting and cleaning, or indoor movement (22). The cognitive function of participants was measured using the Chinese version of the Mini-Mental State Examination (CMMSE) in all waves of the CLHLS (18). The CMMSE consists of 24 items within six dimensions (orientation, registration, naming, attention and calculation, recall, and language). The total score of the CMMSE ranges from 0 to 30 points. Participants with CMMSE scores below 18 points were defined as cognitive impairment as previously validated (18, 23).

### Statistical Analyses

Baseline characteristics of the study population were described as percentages for categorical variables (such as region) and median [interquartile range (IQR)] for continuous variables (such as age). The prevalence of poor quality of life with its 95% *CI* was calculated by sampling weights based on the sampling design. Pearson's χ^2^ test was used to compare the prevalence of poor quality of life in groups with different characteristics. The trends in the prevalence of poor quality of life from 1998 to 2018 were analyzed by the locally weighted scatterplot smoothing regression (LOWESS) analysis and estimated averaged percentage change (EAPC) were estimated (24, 25). We used univariate and multivariate logistic regression models to analyze risk factors related to poor quality of life. Crude odds ratio (COR) and adjusted odds ratio (AOR) with its 95% *CI* was calculated. To examine the robustness of the estimation, we did sensitivity analysis replacing categorical variables with continuous variables, such as age, education level, household income, BMI I, CMMSE scores, and ADL scores. All the analyses were performed with SPSS 26.0 and Stata 17.0. A *p* < 0.05 means significant.

## Results

### Characteristics of the Participants

Of the 91,993 participants included in the study, 73.0% were older than 80 years of age, 52.1% lived in a rural region, 55.8% were women, 81.5% lived with family members, 58.7% never went to school, and 49.2% frequently went to bed hungry in childhood. The age of participants included in the study ranged from 65 to 105 years old, with a median age of 74 years (IQR 69–81). The proportion of current smokers, current drinkers, regular exercise, diverse dietary, and regular organized social activities were 18.0, 20.6, 32.0, 30.4, and 14.8%, respectively. About 30.3% of the participants were underweight, 24.3% had a functional disability, 20.2% had cognitive impairment, 50.5% had self-reported poor or general health, and 42.7% reported unhealthy psychological status (Table 1).

**Table 1:** Prevalence of poor quality of life by the survey year, demographic factors, socioeconomic status, lifestyle habits, and health status (*N* = 91,993).

**Characteristics**	**Total (%)**	**Poor quality of life**		***p*-value**
		**Number**	**Prevalence (%, 95% CI)**	
**Total**	91,993 (100.0)	32,914	38.2 (37.9–38.5)	
**Year**				<0.001^*^
1998	8,460 (9.2)	2,206	27.8 (26.9–28.8)	
2000	10,258 (11.2)	3,354	35.6 (34.7–36.5)	
2002	14,701 (16.0)	5,840	42.8 (42.0–43.6)	
2005	14,320 (15.6)	5,625	42.8 (42.0–43.6)	
2008	14,516 (15.8)	5,908	43.6 (42.9–44.4)	
2011	8,901 (9.7)	3,490	39.2 (38.2–40.2)	
2014	6,492 (7.1)	2,165	33.1 (32.0–34.3)	
2018	14,345 (15.6)	4,326	32.1 (31.4–32.9)	
**Demographic factors**				
Region				<0.001^*^
Urban	43,596 (47.9)	14,120	35.1 (34.6–35.5)	
Rural	47,423 (52.1)	18,478	40.6 (40.2–41.0)	
Gender				0.003^*^
Male	40,677 (44.2)	14,770	38.4 (37.9–38.8)	
Female	51,316 (55.8)	18,144	38.0 (37.6–38.4)	
Age group (years)				<0.001^*^
65–79	24,829 (27.0)	9,850	39.9 (39.5–40.3)	
≥80	67,164 (73.0)	23,064	34.7 (34.1–35.2)	
Marital status				<0.001^*^
Unmarried	987 (1.1)	458	54.0 (51.2–56.8)	
Married	30,904 (33.7)	11,482	37.5 (37.1–37.9)	
Divorced or widowed	59,843 (65.2)	20,878	38.7 (38.2–39.2)	
Living pattern				<0.001^*^
Living with family members	74,671 (81.5)	25,305	36.6 (36.3–37.0)	
Living in an institution	3,282 (3.6)	905	28.0 (26.3–29.8)	
Living alone	13,707 (15.0)	6,585	48.9 (48.1–49.7)	
**Socioeconomic status**				
Years of schooling				<0.001^*^
0	52,571 (58.7)	19,461	40.0 (39.5–40.4)	
≥1	36,999 (41.3)	12,659	36.9 (36.5–37.3)	
Household income per capita				<0.001^*^
Low	38,666 (42.0)	15,756	44.2 (43.7–44.7)	
Moderate	23,925 (26.0)	8,683	38.5 (37.9–39.1)	
High	29,402 (32.0)	8,475	30.4 (29.9–30.9)	
Frequently went to bed hungry in childhood				<0.001^*^
No	46,764 (50.8)	15,836	36.2 (35.8–36.6)	
Yes	45,229 (49.2)	17,078	40.2 (39.8–40.7)	
**Lifestyle habits**				
Smoking status				<0.001^*^
Never	60,361 (65.9)	21,521	37.7 (37.3–38.1)	
Previous	14,711 (16.1)	5,064	37.2 (36.4–38.0)	
Current	16,502 (18.0)	6,186	40.1 (39.4–40.7)	
Alcohol intaking status				0.161
Never	55,967 (67.8)	20,098	37.9 (37.5–38.3)	
Previous	9,544 (11.6)	3,425	37.4 (36.4–38.4)	
Current	17,029 (20.6)	6,249	39.6 (39.0–40.2)	
Regular exercise				<0.001^*^
Never	52,267 (57.2)	20,352	41.0 (40.6–41.4)	
Previous	9,892 (10.8)	3,599	40.2 (39.1–41.4)	
Current	29,206 (32.0)	8,752	33.8 (33.3–34.3)	
Dietary diversity				<0.001^*^
Poor	18,624 (20.3)	8,910	50.4 (49.7–51.2)	
Moderate	45,352 (49.4)	16,010	38.9 (38.5–39.3)	
Good	27,908 (30.4)	7,960	30.9 (30.4–31.4)	
Organized social activities				<0.001^*^
Almost everyday	2,783 (3.0)	756	28.8 (27.5–30.2)	
Sometimes	10,787 (11.8)	3,246	32.8 (32.1–33.5)	
Never	78,172 (85.2)	28,852	39.9 (39.6–40.3)	
**Health status**				
Body mass index (kg/m^2^)				<0.001^*^
Underweight	24,682 (30.3)	9,755	44.2 (43.6–44.9)	
Normal weight	56,734 (69.7)	19,981	37.9 (37.5–38.3)	
Overweight	8,652 (81.8)	2,577	32.0 (31.2–32.7)	
Obesity	1,925 (18.2)	601	31.7 (30.0–33.4)	
Numbers of chronic diseases				<0.001^*^
0	39,424 (43.3)	13,132	34.9 (34.4–35.4)	
1	27,838 (30.5)	9,986	37.7 (37.2–38.3)	
≥2	23,862 (26.2)	9,453	43.6 (43.0–44.2)	
Functional disability				0.001^*^
No	68,780 (75.7)	24,849	37.8 (37.5–38.2)	
Yes	22,037 (24.3)	7,692	41.5 (40.5–42.6)	
Cognitive impairment				<0.001^*^
No	73,380 (79.8)	25,141	37.6 (37.3–37.9)	
Yes	18,613 (20.2)	7,773	48.3 (46.9–49.6)	
Self-reported health				<0.001^*^
Good	45,389 (49.5)	8,012	20.7 (20.3–21.0)	
General	32,687 (35.6)	16,103	52.0 (51.5–52.6)	
Poor	13,687 (14.9)	8,716	67.4 (66.6–68.1)	
Psychological health				<0.001^*^
Healthy	52,741 (57.3)	13,598	29.8 (29.5–30.2)	
Unhealthy	39,252 (42.7)	19,316	53.2 (52.7–53.7)	

* *P < 0.05. Missing data: region 974 (1.1%), marital status 259 (0.3%), living pattern 333 (0.4%), years of schooling 2,423 (2.6%), smoking status 429 (0.5%), alcohol intaking status 9,453 (10.3%), regular exercise 628 (0.7%), dietary diversity 109 (0.1%), organized social activities 251 (0.3%), numbers of chronic diseases 869 (0.9%), functional disability 1,176 (1.3%), and self-reported health 230 (0.3%)*.

### Trends and Disparities in the Prevalence of Poor Quality of Life From 1998 to 2018

**Figure 2:**
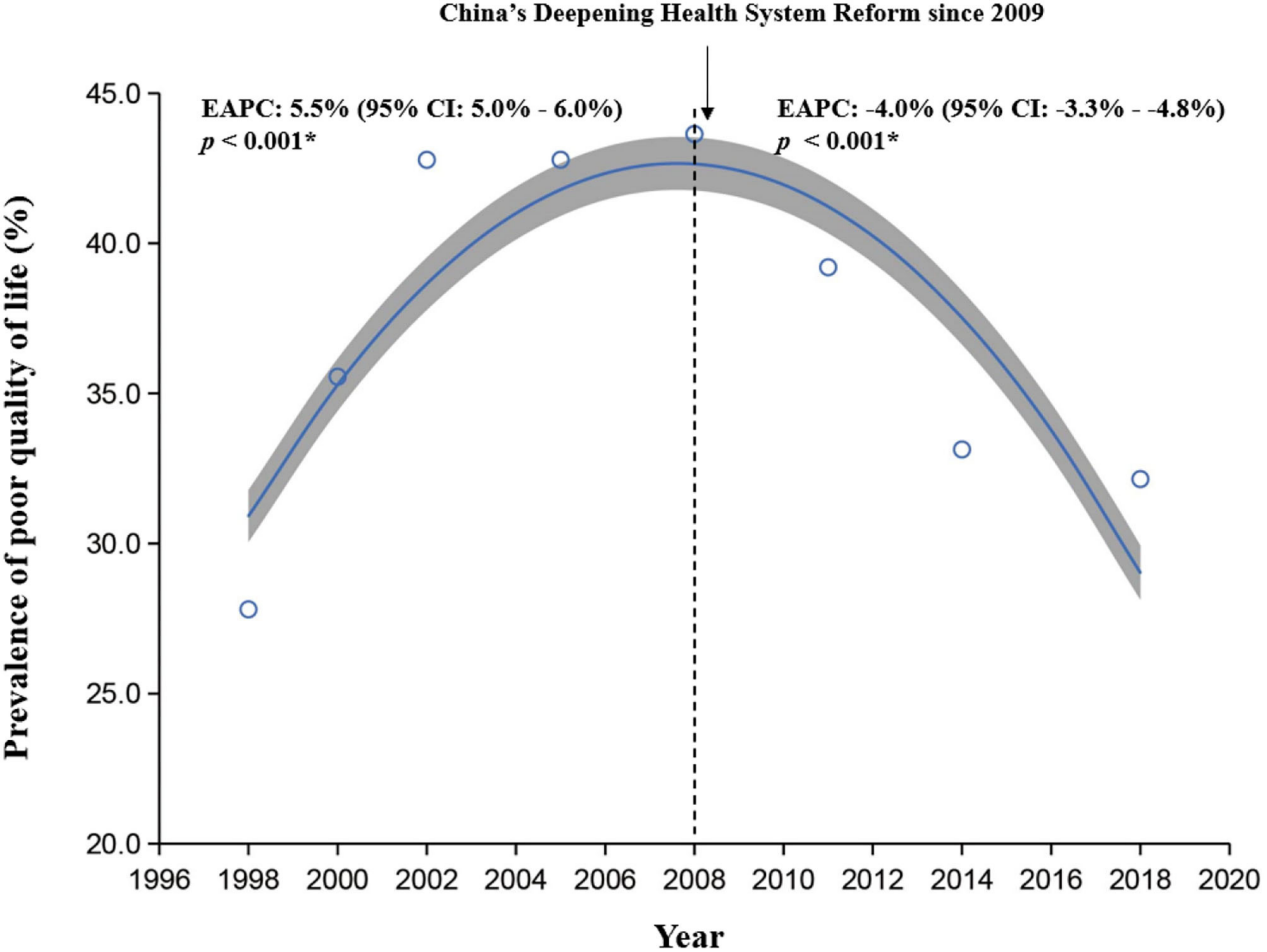
The trends in the prevalence of poor quality of life from 1998 to 2018 by locally weighted scatterplot smoothing regression analysis. EAPC, estimated averaged percentage change.

The prevalence of poor quality of life was 38.2% (95% *CI*: 37.9–38.5%, Table 1). The trends of poor quality of life showed an inverted “U” shape (Figure 2), that the prevalence increased from 27.8% in 1998 to 43.6% in 2008 (EAPC 5.5%, 95% *CI*: 5.0–6.0%), and then decreased from 39.2% in 2011 to 32.1% in 2018 (EAPC −4.0%, 95% *CI*: −3.3 to −4.8%).

**Figure 3:**
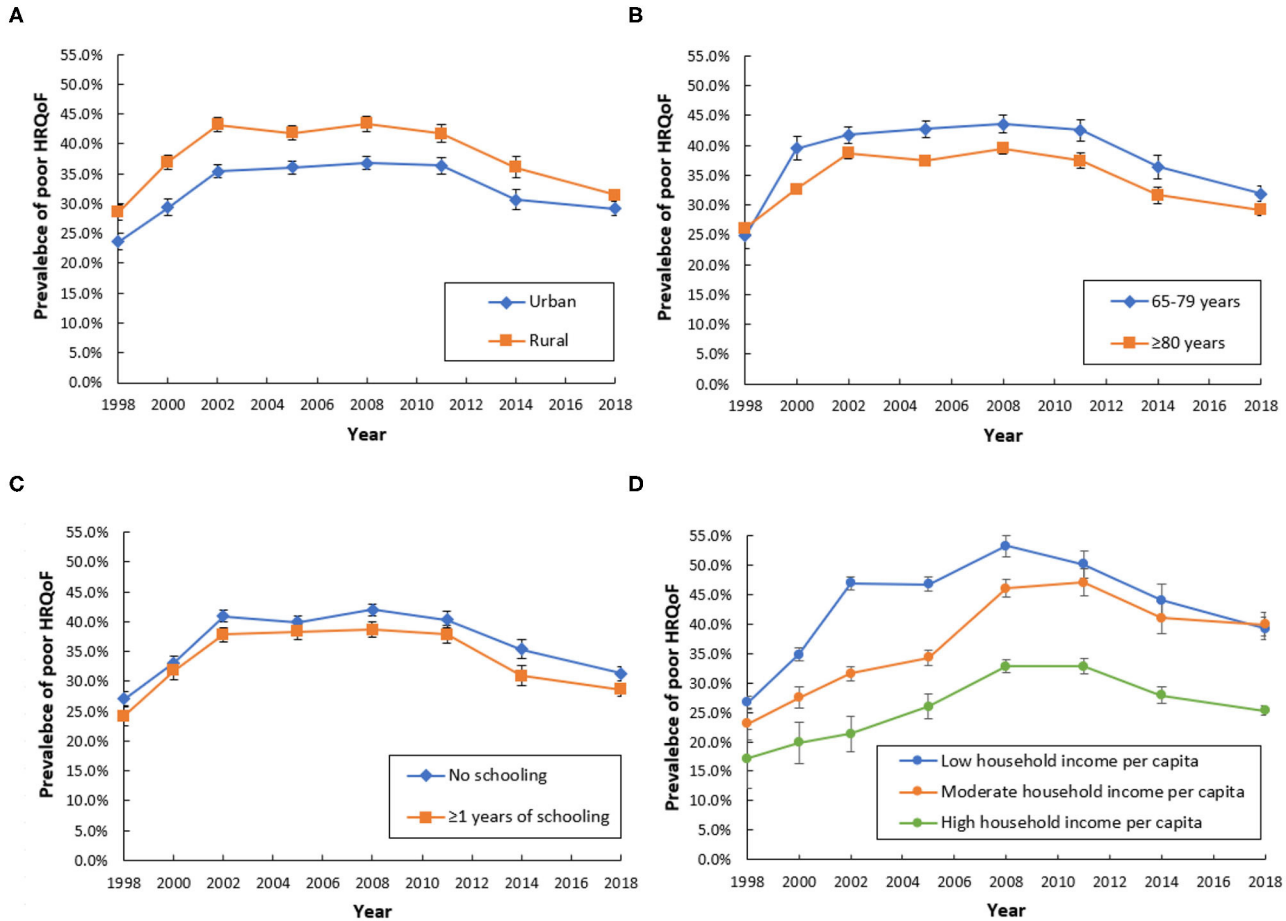
Trends of poor quality of life from 1998 to 2018 by region **(A)**, age **(B)**, education **(C)**, and household income per capita **(D)**.

Similar trends of poor quality of life were observed in rural and urban areas, different age groups, and socioeconomic statuses. However, disparities in the prevalence of poor quality of life were exacerbating among participants with low or moderate household income per capita and participants with high household income per capita from 1998 to 2018 (Figure 3).

In the univariate models, participants living in rural areas, male, with young age, unmarried, living alone, no schooling, low household income, frequently went to bed hungry in childhood, current smoker, current alcohol intaking, no regular exercise, poor dietary diversity, never participating in organized social activities, underweight, with chronic diseases, functional disability, cognitive impairment, poor self-reported health, and unhealthy psychological status were more likely to have a poor quality of life (all *p* < 0.05, (Tables 1, 2).

**Table 2:** Factors related with poor quality of life in the logistic regression models (*N* = 91,993).

**Characteristics**	**Poor quality of life**			
	**Univariate model**		**Multivariate model**	
	**cOR (95% CI)**	***p*-value**	**aOR (95% CI)**	***p*-value**
**Demographic factors**				
Year	0.993 (0.995–0.991)	<0.001^*^	0.99 (0.98–0.99)	<0.001^*^
Region				
Urban	1		1	
Rural	1.11 (1.16–1.07)	<0.001^*^	1.18 (1.12–1.24)	<0.001^*^
Gender				
Male	1			
Female	0.98 (1.01–0.96)	0.245		
Age group (years)				
65–79	1.25 (1.29–1.22)	<0.001^*^	1.75 (1.65–1.86)	<0.001^*^
≥80	1		1	
Marital status				
Unmarried	1.96 (2.20–1.75)	<0.001^*^	1.66 (1.32–2.09)	<0.001^*^
Divorced or widowed	1		1	
Married	1.06 (1.08–1.03)	<0.001^*^	1.00 (0.95–1.06)	0.988
Living pattern				
Living with family members	1.48 (1.62–1.36)	<0.001^*^	1.95 (1.67–2.28)	<0.001^*^
Living in an institution	1		1	
Living alone	2.46 (2.69–2.24)	<0.001^*^	3.01 (2.56–3.55)	<0.001^*^
**Socioeconomic status**				
Years of schooling				
0	1			
≥1	1.14 (1.17–1.11)	<0.001^*^		
Household income per capita				
Low	1.82 (1.87–1.76)	<0.001^*^	1.53 (1.42–1.64)	<0.001^*^
Moderate	1.43 (1.48–1.38)	<0.001^*^	1.33 (1.25–1.42)	<0.001^*^
High	1		1	
Frequently went to bed hungry in childhood				
No	1			
Yes	1.19 (1.23–1.15)	<0.001^*^		
**Lifestyle habits**				
Smoking status				
Never	1		1	
Previous	0.98 (1.01–0.94)	0.220	1.04 (0.97–1.11)	0.291
Current	1.10 (1.14–1.07)	<0.001^*^	1.24 (1.17–1.31)	<0.001^*^
Alcohol intaking status				
Never	1			
Previous	0.98 (1.03–0.93)	0.368		
Current	1.07 (1.11–1.04)	<0.001^*^		
Regular exercise				
Never	1.36 (1.40–1.32)	<0.001^*^		
Previous	1.32 (1.39–1.25)	<0.001^*^		
Current	1			
Dietary diversity				
Poor	2.27 (2.36–2.19)	<0.001^*^	1.72 (1.60–1.85)	<0.001^*^
Moderate	1.42 (1.46–1.38)	<0.001^*^	1.27 (1.21–1.34)	<0.001^*^
Good	1		1	
Organized social activities				
Almost everyday	1		1	
Sometimes	1.21 (1.30–1.12)	<0.001^*^	1.34 (1.19–1.50)	<0.001^*^
Never	1.64 (1.76–1.54)	<0.001^*^	1.41 (1.27–1.57)	<0.001^*^
**Health status**				
Body mass index (kg/m^2^)
Underweight	1.30 (1.34–1.25)	<0.001^*^	1.00 (0.94–1.07)	0.898
Normal weight	1		1	
Overweight	0.77 (0.80–0.74)	<0.001^*^	0.87 (0.82–0.92)	<0.001^*^
Obesity	0.76 (0.82–0.70)	<0.001^*^	0.84 (0.74–0.94)	0.003^*^
Numbers of chronic diseases				
0	1		1	
1	1.13 (1.17–1.10)	<0.001^*^	1.11 (1.03–1.19)	<0.001^*^
≥2	1.44 (1.49–1.39)	<0.001^*^	1.15 (1.08–1.22)	<0.001^*^
Functional disability				
No	1		1	
Yes	1.17 (1.22–1.12)	<0.001^*^	1.33 (1.23–1.45)	<0.001^*^
Cognitive impairment				
No	1			
Yes	1.55 (1.64–1.47)	<0.001^*^		
Self-reported health				
Good	1		1	
General	4.16 (4.29–4.04)	<0.001^*^	4.06 (3.86–4.27)	<0.001^*^
Poor	7.93 (8.26–7.60)	<0.001^*^	6.83 (6.32–7.38)	<0.001^*^
Psychological health				
Healthy	1		1	
Unhealthy	2.68 (2.75–2.60)	<0.001^*^	1.90 (1.81–2.00)	<0.001^*^

*COR, crude odds ratio; AOR, adjusted odds ratio*.

* *p < 0.05*.

### Factors Related to Poor Quality of Life in the Multivariate Model

After controlling potential confounders, living in rural areas (AOR 1.18, 95% *CI*: 1.12–1.24), aged below 80 years (AOR 1.75, 95% *CI*: 1.65–1.86), unmarried (AOR 1.66, 95% *CI*: 1.32–2.09), living alone (AOR 3.01, 95% *CI*: 2.56–3.55), low household income (AOR 1.53, 95% *CI*: 1.42–1.64), current smoker (AOR 1.24, 95% *CI*: 1.17–1.31), poor dietary diversity (AOR 1.72, 95% *CI*: 1.60–1.85), never participated in organized social activities (AOR 1.41, 95% *CI*: 1.27–1.57), with chronic diseases (AOR 1.15, 95% *CI*: 1.08–1.22), functional disability (AOR 1.33, 95% *CI*: 1.23–1.45), poor self-reported health (AOR 6.83, 95% *CI*: 6.32–7.38), and unhealthy psychological status (AOR 1.90, 95% *CI*: 1.81–2.00) were risk factors related with the poor quality of life in the multivariate model (all *p* < 0.05, Table 2). In the sensitivity analysis, the results were stable.

## Discussion

To our knowledge, this was the first study that assessed the 20-year trends and disparities in quality of life among older adults in China from 1998 to 2018. In the present study, we found that overall, more than one-third of the older adults (38.2%) perceived poor quality of life in the past two decades, and the time trends of poor quality of life showed an inverted “U” shape, with an increasing trend during 1998–2008 and a decreasing trend during 2009–2018. China started the first round of health system reform in 1996, but the effectiveness of the reform was questioned after several years of implementation for complaints from the public about access to and affordability of healthcare increased (26). According to the national health services survey in 2008, a large proportion of the population in China could not afford the needed healthcare (26, 27). As the outbreak of severe acute respiratory syndrome (SARS) in 2003 in China highlighted the importance of health for human development, the Chinese government began to recognize the contribution of the health system to entire social and economic development and started to plan another round of health system reform in 2007 (26). The failure of the first round of health system reform might be one possible explanation for the increasing trends of poor quality of life.

The decreasing trend of quality of life after 2008 might be related to the achievement of China's deepening health system reform on healthcare since 2009 (26). After 2008, the Central Committee of the Communist Party of China and the State Council issued the Opinions on Deepening Health System Reform in 2009, with the aim of establishing an equitable and effective health system for all people (universal health coverage) by 2020, by strengthening the provision of essential medicines, healthcare delivery, and health security (26, 28). Since 2009, China's deepening health system reform on healthcare was conducted. Yao et al. compared the 2008 and 2013 national health services surveys in China and reported decreased problems in the quality of life (14). Our findings were consistent with the results in the general population.

It is well-known that subjective quality of life is a construct determined by multiple factors (29). Sociodemographic, physical, and psychological factors could influence the subjective quality of life (7). In the multivariate model, we found that aged below 80 years, living in rural areas, unmarried, living alone, low household income, current smoker, poor dietary diversity, never participated in organized social activities, with chronic diseases, functional disability, poor self-reported health, unhealthy psychological status were risk factors related with the poor quality of life, after controlling potential confounders. The findings were consistent with previous studies (2, 4, 5, 7). It is well-reported that cognition and behavior change significantly, along with the associated brain function and organization as humans age (30). Age-dependent physical and psychological dependence might be related to the poor quality of life (31). Living alone, smoking habits, poor dietary diversity, and social isolation were all modifiable risk factors, which could be intervened.

We found that the quality of life in participants with low socioeconomic status was much worse than participants with high socioeconomic status, which was consistent with previous studies (7, 32). Previous studies had reported that differences in socioeconomic status were a significant factor in explaining the different health statuses among the Chinese older people (32–34). The relationship between socioeconomic status and quality of life might be explained by the difference in living standards and the access to healthcare services (12). Gross domestic product (GDP) per capita has increased more than 10 times in China in the past 20 years, from $ 828.58 in 1998 to $ 9,976.68 in 2018. However, we found that disparities in the prevalence of poor quality of life were exacerbating among participants with different socioeconomic statuses, despite the rapid development of China's socioeconomic level in the past two decades. The underlying reason behind this phenomenon is worthy of further research in the future.

As for practical recommendations, our findings highlighted the importance of strengthening the health system on improving the multidimensional quality of life among older adults. More operationalized policies are needed to improve the current situation of health inequities among the different socioeconomic statuses in China and to address the health equity problem in a more systematic way. Currently, insufficient integration of medical treatment and public health hampered the equity of health, equity of health service utilization, and equity of accessibility of older adults in China. Chinese health departments should take advantage of China's development paradigm shift in recent years from efficiency-orientation to sustainability and equality-orientation in the construction of Healthy China 2030. Quality of life should also be added as one of the main health indicators in monitoring the progress in the Healthy China 2030 plan.

There were several limitations in this study. First, the measurement of quality of life was not based on complex tools, such as SF-36, because of the original study design on the questionnaire in the CLHLS. Second, recall bias could not be avoided when we did the survey among older adults, compared with youngsters. Third, some confounding factors (e.g., major life stress events) may affect the quality of life. However, the CLHLS did not collect the information on these potential confounders. Thus, we could not include these factors in the multivariable models. Finally, the results of this study only represent the quality of life of the elderly population in mainland China. In the future, it is necessary to carry out longitudinal tracking of quality of life around the world and carry out multi-country comparative studies.

In conclusion, the prevalence of poor quality of life in Chinese older adults showed an inverted “U” trend from 1998 to 2018 during the past two decades. It peaked in 2008 and declined since China's deepening health system reform in 2009. However, disparities in the poor quality of life were exacerbating among participants with different socioeconomic statuses. Quality of life was affected by physical—psychological—social multidimensional factors. Strengthening the health system is of great importance in improving the quality of life. More efforts are needed to promote quality of life among Chinese older adults, especially for reducing the disparities in quality of life among population different socioeconomic levels.

## Data Availability Statement

The original contributions presented in the study are included in the article/Supplementary Material, further inquiries can be directed to the corresponding authors.

## Ethics Statement

The studies involving human participants were reviewed and approved by the Ethical Review Committee of Peking University (IRB00001052-13074). The patients/participants provided their written informed consent to participate in this study.

## Author Contributions

JL contributed to the conceptualization, formal analysis, writing the original draft, writing review and editing, and funding acquisition. JW contributed to the writing review and editing. All the authors have made substantial contributions to the conception, design of the study, or the acquisition, analysis, or interpretation of data for the study. They have participated in drafting the manuscript and approval of the version to be published.

## Funding

This study was supported by the National Natural Science Outstanding Youth Foundation of China (Grant Numbers: 72122001) and the National Natural Science Foundation of China (Grant Numbers: 72042002 and 72061160491). The funding body had no role in the design or conduct of the study; the collection, management, analysis, or interpretation of the data; the preparation, review, or approval of the manuscript; or the decision to submit the manuscript for publication.

## Conflict of Interest

The authors declare that the research was conducted in the absence of any commercial or financial relationships that could be construed as a potential conflict of interest.

## Publisher's Note

All claims expressed in this article are solely those of the authors and do not necessarily represent those of their affiliated organizations, or those of the publisher, the editors and the reviewers. Any product that may be evaluated in this article, or claim that may be made by its manufacturer, is not guaranteed or endorsed by the publisher.
