# Supplementary material for: Trends and Disparities in Quality of Life Among Older Adults From 1998 to 2018 in China: A National Observational Study
Source: Front Med (Lausanne). 2022 Jan 28;8:796208. doi: 10.3389/fmed.2021.796208 (PMC8832120; doi:10.3389/fmed.2021.796208)
Supplement: Supplementary file 1 [file Table_1.DOCX]

**Supplemental Table 1. Prevalence of poor quality of life in different demographic factors stratified by the survey year (N=91993)**

| Demographic factors | | Poor quality of life (%) | | | | | | | |
| --- | --- | --- | --- | --- | --- | --- | --- | --- | --- |
|  |  | 1998 | 2000 | 2002 | 2005 | 2008 | 2011 | 2014 | 2018 |
| Region | Urban | 1208 (25.5) | 1102 (30.8) | 2026 (37.0) | 2729 (40.2) | 2774 (40.4) | 1436 (39.1) | 799 (29.9) | 2485 (32.4) |
|  | Rural | 1205 (30.6) | 2700 (38.0) | 4665 (45.9) | 4069 (44.7) | 4169 (46.1) | 2191 (39.3) | 1114 (36.5) | 2388 (32.0) |
| Gender | male | 899 (27.6) | 1540 (38.3) | 3133 (42.6) | 3253 (42.7) | 3279 (42.9) | 1745 (38.2) | 1030 (32.3) | 2366 (32.4) |
|  | female | 1513 (27.9) | 2262 (33.9) | 3558 (43.0) | 3545 (42.8) | 3664 (44.3) | 1882 (40.2) | 1136 (34.0) | 2530 (31.9) |
| Age group (years) | 65-79 | 0 (0.0) | 0 (0.0) | 5802 (42.8) | 5896 (43.0) | 5858 (43.8) | 2946 (38.9) | 1679 (33.1) | 4027 (32.4) |
|  | >=80 | 2412 (27.8) | 3802 (35.6) | 889 (42.4) | 902 (41.7) | 1085 (42.7) | 681 (40.5) | 487 (33.2) | 869 (31.1) |
| Marital status | Unmarried | 49 (46.5) | 53 (40.4) | 128 (57.9) | 89 (58.1) | 116 (69.2) | 58 (34.8) | 68 (66.6) | 90 (56.9) |
|  | Married | 625 (26.0) | 1152 (37.9) | 3842 (40.7) | 4085 (41.3) | 4311 (43.0) | 2325 (39.3) | 1150 (27.8) | 3297 (30.9) |
|  | Divorced or widowed | 1733 (28.1) | 2598 (34.5) | 2721 (45.4) | 2624 (44.9) | 2516 (44.0) | 1239 (39.4) | 925 (41.6) | 1466 (34.5) |
| Living pattern | Living with family members | 1788 (25.7) | 2840 (34.3) | 5442 (40.9) | 5635 (41.2) | 5667 (42.1) | 2933 (38.6) | 1530 (29.6) | 3900 (30.6) |
|  | Living in a institution | 74 (12.8) | 196 (23.4) | 136 (37.2) | 66 (33.6) | 43 (34.7) | 31 (37.7) | 89 (54.8) | 96 (36.9) |
|  | Living alone | 551 (47.7) | 767 (48.3) | 1113 (56.4) | 1094 (54.4) | 1233 (52.7) | 619 (41.0) | 530 (46.1) | 823 (40.5) |
